# Decision-making process of exclusive breastfeeding at 6 months postpartum based on the COM-B model: a multicenter, cross-sectional study in Southeast China

**DOI:** 10.3389/fpubh.2026.1859936

**Published:** 2026-09-04

**Authors:** Wu-lan Wang, Bao-ling Gao, Jing Zeng, Gui-hua Liu, Ying-ping Huang, Xiao-feng Zhang, Xiao-qiu Guo, Sheng-bin Guo

**Affiliations:** 1 Fujian Maternity and Child Health Hospital College of Clinical Medicine for Obstetrics & Gynecology and Pediatrics, Fujian Medical University, Fujian Province, China; 2 Fujian Obstetrics and Gynecology Hospital, College of Clinical Medicine for Obstetrics & Gynecology and Pediatrics, Fujian Medical University, Fuzhou, Fujian Province, China

**Keywords:** 6 months postpartum, COM-B model, cross-sectional study, exclusive breastfeeding, structural equation modeling

## Abstract

**Background:**

Exclusive breastfeeding (EBF) for the first 6 months is crucial for public health, yet EBF rates in China remain below the 50% target of “Healthy China 2030.” In Southeast China, adequate maternal knowledge and healthcare access do not ensure sustained EBF practices, indicating a clear knowledge–behavior gap. Traditional education models often ignore the interaction between psychological capabilities and environmental opportunities. Grounded in the COM-B (Capability, Opportunity, Motivation-Behavior) model, this multicenter study investigates EBF decision-making among postpartum women in this region. By examining how Capability (knowledge) and Opportunity (social support) interact through Motivation (self-efficacy), this research provides an evidence-based framework for targeted EBF interventions.

**Methods:**

A multicenter, cross-sectional study was conducted. We used convenience sampling to recruit 400 women at 6 months postpartum from two public hospitals in Southeast China. Validated instruments, including the Breastfeeding Knowledge Questionnaire, Breastfeeding Family Support Scale, and Breastfeeding Self-Efficacy Scale (Short Form), were used for data collection. Data analysis involved descriptive statistics, independent t-tests, ANOVA, and Partial Least Squares Structural Equation Modeling (PLS-SEM) to test the hypothesized pathways.

**Results:**

The exclusive breastfeeding (EBF) rate at 6 months postpartum was 30.25%. The PLS-SEM model explained 35.7% of the variance in EBF behavior (*R*^2^ = 0.357). Breastfeeding knowledge (Capability)did not directly predict EBF behavior (*p* = 0.534). However, it showed a significant indirect effect. Specifically, knowledge positively predicted self-efficacy (Motivation) (*B* = 0.136), which in turn predicted EBF (*B* = 0.201). Furthermore, family support (Opportunity) had a direct positive effect on EBF (*B* = 0.097). It also predicted self-efficacy (*B* = 0.528), demonstrating a significant indirect effect on EBF behavior.

**Conclusion:**

Based on the COM-B model, this study identifies maternal self-efficacy (Motivation) as a key mediator connecting breastfeeding knowledge (Capability) and family support (Opportunity) to EBF behavior. These findings suggest that knowledge-focused education alone is insufficient. Future public health interventions should adopt a family-centered approach. Clinical programs should integrate family support and prioritize building maternal confidence to effectively improve EBF rates.

## 1 Background

Exclusive breastfeeding (EBF) is a cornerstone of global public health. Its benefits extend beyond maternal and child health to include economic and environmental advantages (1). For infants, breast milk provides irreplaceable “triple protection” by integrating nutritional, immunological, and microbial regulation. This dynamic fluid significantly reduces the risk of early childhood infections (e.g., diarrhea, respiratory infections) and sudden infant death syndrome. It also offers long-term protection against chronic conditions like inflammatory bowel disease, childhood leukemia, and metabolic syndrome (2–4). Furthermore, a longer breastfeeding duration positively impacts neurodevelopment, leading to enhanced cognitive function and higher IQ scores (5). Breastfeeding also strongly protects maternal health. It facilitates postpartum uterine involution through neuroendocrine regulation. Additionally, it significantly lowers the long-term risks of breast cancer, ovarian cancer, type 2 diabetes, and cardiovascular diseases (6, 7). Notably, it offers psychological and autoimmune benefits. For instance, EBF is associated with a 53% lower risk of postpartum depression (8) and a 32% lower incidence of rheumatoid arthritis (9).

Beyond individual health, breastfeeding has profound societal impacts. Suboptimal breastfeeding practices impose a heavy global economic burden (10). Given these broad advantages, promoting breastfeeding is more than just a clinical recommendation. It is a strategic public health imperative aligned with the United Nations Sustainable Development Goals (SDGs 3, 12, and 13). Therefore, the World Health Organization (WHO) and the United Nations Children’s Fund (UNICEF) strongly advocate for EBF during the first 6 months of life as the global gold standard for infant feeding (11).

Despite sustained policy support and health education initiatives, postpartum mothers continue to face substantial challenges in maintaining exclusive breastfeeding (EBF) practices (12). In fact, our previous multi-center investigation in Southeast China revealed that the EBF rate drops to merely 44.2% as early as 42 days (6 weeks) postpartum (13). This precipitous decline, occurring long before the WHO-recommended 6-month mark, underscores a persistent “intention-behavior gap” in maternal feeding practices that warrants deeper investigation.

Existing research has systematically examined breastfeeding-related barriers, generally categorizing these determinants into three principal dimensions: (1) Individual-level factors, encompassing maternal age, occupation, educational attainment, breastfeeding knowledge, and maternal self-efficacy (14–16); (2) Household-level factors, including spousal support, household socioeconomic status, and overall familial support systems (17–19); and (3) Societal-environmental factors, comprising social policies, employment conditions, and community support networks (18, 20).

While these determinants have been extensively investigated, critical research gaps persist. First, previous studies frequently relied on traditional behavioral models, such as the Theory of Planned Behavior (TPB) or the Health Belief Model (HBM) (21). These models primarily focus on individual cognitive factors and intentions. However, they often fail to account for external environmental constraints and actual skill proficiency (22). Consequently, they struggle to fully explain the well-documented “intention-behavior gap” in breastfeeding practices. Second, empirical findings remain inconsistent regarding the actual predictive power of breastfeeding knowledge (23) and social support (24). Most notably, previous research mainly examined isolated factors. There is a lack of systematic analysis regarding their complex interactions.

To disentangle these complex decision-making mechanisms, this study employs the Capability, Opportunity, Motivation-Behavior (COM-B) model (25) as its theoretical foundation. This framework offers a meaningful advancement over prior models. Moving beyond traditional cognitive paradigms, the COM-B model posits that behavior is generated by a dynamic, synergistic interplay among intrinsic capabilities, external environmental opportunities, and internal motivational drives (26). It integrates both internal psychology and external systems to address the limitations of older models. Furthermore, conducting a mediation analysis is particularly necessary in this context. Previous studies often simply tested whether knowledge or support directly predicted breastfeeding outcomes. However, possessing breastfeeding knowledge (Capability) or receiving family support (Opportunity) may not automatically translate into sustained behavior. Instead, we hypothesize that these resources serve as a foundation to build a mother’s self-efficacy (Motivation) (27). Therefore, testing these mediational pathways allows us to formally examine whether motivation bridges the gap between baseline resources and actual practice. While this comprehensive framework has been leveraged to analyze other health-related behaviors (28–30) its mediational mechanisms in the context of prolonged exclusive breastfeeding remain systematically underexplored.

In this study, we operationalize the three core dimensions of the COM-B model to reflect the specific realities of postpartum women in Southeast China:

Capability (breastfeeding knowledge): This dimension encompasses the knowledge base and psychological capacity required to perform a behavior. Here, it is operationalized as maternal breastfeeding knowledge (31). We hypothesize that while knowledge provides the foundational capability, its direct impact on sustained behavior may be limited unless effectively channeled through internal motivation.

Opportunity (family support): Opportunity comprises factors outside the individual that facilitate behavior. Given the profound influence of traditional family dynamics in Southeast China, we focus on family support as the critical indicator of social opportunity. While general social support sometimes yields inconsistent associations (24), targeted familial reinforcement significantly mitigates environmental barriers and improves breastfeeding continuity (13).

Motivation (breastfeeding self-efficacy): The motivation dimension incorporates both automatic processes and reflective evaluations (25). We conceptualize this as breastfeeding self-efficacy—a mother’s perceived confidence in her ability to sustain breastfeeding amidst challenges (32). Recognized as a paramount predictor of breastfeeding duration (33), self-efficacy serves as the reflective motivational engine.

**Figure 1:**
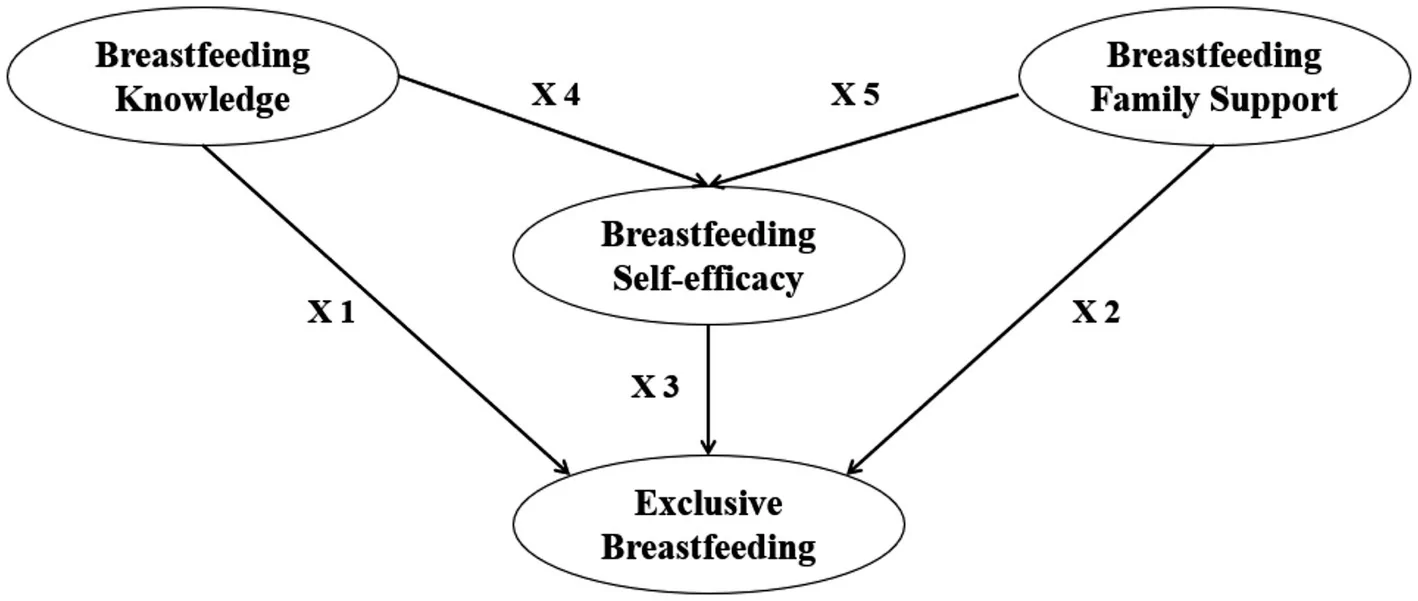
Theoretical model and hypotheses.

Based on the theoretical framework of the COM-B model and existing empirical literature, this study proposes the following five hypotheses regarding exclusive breastfeeding (EBF) behavior at 6 months postpartum, as illustrated in Figure 1:

*H1*: Maternal breastfeeding knowledge (Capability) exerts a direct positive effect on EBF behavior.

*H2*: Family support (Opportunity) exerts a direct positive effect on EBF behavior.

*H3*: Breastfeeding self-efficacy (Motivation) exerts a direct positive effect on EBF behavior.

*H4*: Breastfeeding self-efficacy (Motivation) mediates the relationship between breastfeeding knowledge and EBF behavior.

*H5*: Breastfeeding self-efficacy (Motivation) mediates the relationship between family support and EBF behavior.

In summary, the individual impacts of breastfeeding knowledge, family support, and self-efficacy on EBF are widely documented. However, their structural interdependencies and collective mechanisms remain underexplored. Specifically, empirical evidence within the socio-cultural context of Southeast China is lacking. Few studies utilize a comprehensive behavioral framework to decode the complex transition from maternal intention to actualized exclusive breastfeeding behavior at 6 months postpartum.

To bridge this critical gap, we conducted a multi-center, cross-sectional study based on the COM-B model. We aimed to systematically investigate the decision-making process of EBF at 6 months postpartum. By utilizing Partial Least Squares Structural Equation Modeling (PLS-SEM), we mapped and quantified the complex pathways among Capability (knowledge), Opportunity (family support), Motivation (self-efficacy), and final EBF Behavior. Ultimately, our findings aim to provide a robust, evidence-based framework for public health strategies. We recommend shifting from traditional, unidirectional knowledge education toward “family-centered” empowerment interventions. This shift can effectively bridge the intention-behavior gap and optimize maternal and child health outcomes.

## 2 Materials and methods

### 2.1 Study design and participants

We conducted a multi-center, cross-sectional descriptive survey between December 2024 and May 2025. Participants were recruited using convenience sampling. A total of 400 mothers at 6 months postpartum were included from two public hospitals in Southeast China. The inclusion criteria were as follows: (1) maternal age ≥ 20 years; (2) exactly 6 months postpartum; (3) normal cognitive ability; and (4) voluntary participation.

We acknowledge that recruiting from hospital clinics may introduce selection bias. Specifically, these mothers may have better healthcare access and higher breastfeeding awareness compared to the broader postpartum population.

The sample size was calculated by PASS 16. Based on the reported exclusive breastfeeding rate of 36.2% among infants within 6 months in China (34), Zα = 1.96 (corresponding to a 95% confidence level), *p* = 0.362 (expected prevalence rate), and *d* = 0.05(margin of error). The initial calculation yielded a minimum sample size of 354 participants. After a nonresponse rate of 10% was considered, the minimum sample size was 389 participants.

### 2.2 Ethics approval and consent to participate

The study was approved by the Ethical Committee of Fujian Maternity and Child Health Hospital College of Clinical Medicine for Obstetrics and Gynecology and Pediatrics, Fujian Medical University, China (No. 2024KY277). The study was conducted in accordance with the Helsinki Declaration, and written and verbal informed consent to participate was obtained.

### 2.3 Measures

#### 2.3.1 The socio-demographic characteristics

The questionnaire gathered the postpartum mothers within 6 months data: includes maternal age, education level, employment status, family income, BMI index, maternity leave duration, delivery method, their family’s breastfeeding advice, whether Mother and infant rooming together, etc.

#### 2.3.2 Feeding method

The infant feeding method was determined by asking the mothers at 6 months postpartum what they fed their babies in the last 24 h and what they usually feed them. Exclusive breastfeeding was defined as no food or liquid other than breast milk (not even water) given to infants (11). Mixed feeding (11) refers to adding formula and other dairy products to infants in addition to breast milk. Artificial feeding (11) refers to feeding infants solely with foods other than breastfeeding.

#### 2.3.3 Breastfeeding knowledge questionnaire

The questionnaire developed by Min (35), is utilized to assess maternal breastfeeding knowledge. The breastfeeding knowledge questionnaire consists of 17 items organized into two subscales: breastfeeding benefits and breastfeeding skills. Each item was dichotomously scored, with correct responses assigned a value of 1 and incorrect responses assigned a value of 0, yielding a total possible score range of 0–17. Higher total scores reflect a greater level of breastfeeding knowledge among participants. The content validity of the questionnaire is 0.91, and in the current study, Cronbach’s alpha value was 0.953.

#### 2.3.4 Breastfeeding family support questionnaire

This questionnaire developed by Zhu et al. (36), is utilized to assess perceived family support for breastfeeding. The instrument comprises two distinct dimensions: psychological support (items 1–7) and behavioral support (items 8–9). All items are self-reported measures utilizing a 4-point Likert-type scale (ranging from 1 to 4). The total family support score is calculated as the mean score across all nine items, with score interpretations as follows: a score of 1 represents low family support, scores between 2 and 3 indicate moderate family support, and a score of 4 reflects high family support. Cronbach’s alpha value was 0.886.

2.3.5 Breastfeeding Self-Efficacy Scale Short Form

The Breastfeeding Self-Efficacy Scale-Short Form, originally developed by Dennis (37), is a psychometric instrument designed to evaluate maternal breastfeeding self-efficacy. This 14-item scale yields a total score ranging from 14 to 70, with higher scores indicating greater levels of breastfeeding self-efficacy. The psychometric properties of the Chinese version of the BSES-SF were established by Liu et al. (38), demonstrating strong reliability with a Cronbach’s alpha coefficient of 0.927. In the current study, Cronbach’s alpha value was 0.963.

### 2.4 Data collection

A web-based survey was distributed via the Wen Juan Xing platform (39), which is one of the leading professional and free online survey platforms in China. Prior to launching the survey, permission was obtained from hospital administrators. Researchers then generated a quick response (QR) code for the survey through Wen Juan Xing. Data collectors recruited participants from the postpartum clinic based on the study’s inclusion and exclusion criteria. They also provided an explanation of the study’s purpose and significance, ensuring adherence to the principles of voluntariness, anonymity, and confidentiality to secure participants’ cooperation. Participants could scan the QR code using WeChat to access and complete the survey online. It is important to note that the Wen Juan Xing platform allowed only one submission per WeChat account, and incomplete or missing data could not be submitted.

### 2.5 Statistical analysis

The data was organized using EpiData 3.1 software for double entry, and IBM SPSS27.0 software for descriptive statistical analysis, independent sample t-test, one-way ANOVA, and Pearson correlation analysis. Using Smartpls 4.0 software to construct a Partial Least Squares Structural Equation Model (PLS-SEM model), parameter estimation using Partial Least Squares method, with Exclusive Breastfeeding Behavior as the outcome, Breastfeeding knowledge and Breastfeeding Family Support as independent variables, and Breastfeeding Self-Efficacy as the mediating variable to calculate the mediating effect of Breastfeeding Self-Efficacy, with double-sided *p* < 0.05 indicates a statistically significant difference. The statistical significance of the PLS-SEM estimates was evaluated using the nonparametric bootstrapping procedure in SmartPLS. A total of 5,000 bootstrap subsamples were generated. Standard errors, *t*-values, *p*-values, and 95% confidence intervals were obtained for the path coefficients, indirect effects, and measurement model parameters. A two-tailed test was applied, and *p* < 0.05 was considered statistically significant.

## 3 Results

### 3.1 Relationship between maternal socio-demographic data and exclusive breastfeeding behavior

A total of 400 questionnaires were completed by mothers at 6 months postpartum through convenience sampling across multiple hospitals. Among these participants, 121 mothers (30.25%) reported practicing exclusive breastfeeding for their infants. Additional maternal socio-demographic characteristics are summarized in Table 1. The majority of the mothers were between 31 and 40 years of ages (55.0%). Nearly half of the participants have obtained a bachelor’s degree (46.5%) and more than half of them work in fixed occupation (62.25%). One-way ANOVA revealed statistically significant differences in feeding patterns based on several factors: parity (number of children), family feeding recommendations and room-sharing practices between mother and infant.

**Table 1:** General characteristics of subjects (*N* = 400).

Variables	Categories	EBF (*N* = 121)	N-EBF(*N* = 279)	*χ* ^2^ */t*	*P*
Age(years)	18 ~ 25	5.00(4.13%)	21.00(7.53%)	–	0.340
	26 ~ 30	41.00(33.88%)	109.00(39.07%)		
	31 ~ 40	74.00(61.16%)	146.00(52.33%)		
	41 ~ 50	1.00(0.83%)	3.00(1.07%)		
Maternal education level	High school and below	22.00(18.18%)	59.00(21.15%)	2.540	0.470
	Vocational college	32.00(26.45%)	82.00(29.39%)		
	Undergraduate	63.00(52.07%)	123.00(44.09%)		
	Master’s degree and above	4.00(3.30%)	15.00(5.37%)		
Occupational type	Fixed occupation	75.00(61.98%)	174.00(62.37%)	2.050	0.360
	Freelance occupation	20.00(16.53%)	33.00(11.83%)		
	unemployed	26.00(21.49%)	72.00(25.80%)		
Monthly income (yuan)	<3,000	4.00(3.31%)	10.00(3.58%)	–	0.590
	3,000 ~ 5,999	36.00(29.75%)	71.00(25.45%)		
	6,000 ~ 9,999	44.00(36.36%)	121.00(43.37%)		
	≥10,000	37.00(30.58%)	77.00(27.60%)		
BMI	<18.5	6.00(4.96%)	18.00(6.45%)	1.330	0.720
	18.5 ~ 23.9	84.00(69.42%)	199.00(71.33%)		
	24 ~ 28	26.00(21.49%)	48.00(17.20%)		
	>28	5.00(4.13%)	14.00(5.02%)		
Maternity leave duration	<6 months	39.00(32.23%)	89.00(31.90%)	2.880	0.410
	6 months	41.00(33.88%)	102.00(36.56%)		
	>6 months	9.00(7.44%)	10.00(3.58%)		
	Unlimited	32.00(26.45%)	78.00(27.96%)		
Mode of delivery	Vaginal delivery	46.00(38.02%)	109.00(39.07%)	1.060	0.590
	Vaginal Delivery (With Episiotomy)	32.00(26.44%)	61.00(21.86%)		
	Cesarean section	43.00(35.54%)	109.00(39.07%)		
Parity	1	73.00(60.33%)	199.00(71.33%)	–	0.026
	2	41.00(33.88%)	75.00(26.88%)		
	≥3	7.00(5.79%)	5.00(1.79%)		
Family recommendations on feeding methods	Exclusive breastfeeding	114.00(94.21%)	66.00(23.66%)	170.000	<0.001
	Mixed feeding	7.00(5.79%)	157.00(56.27%)		
	Artificial feeding	0.00(0.00%)	56.00(20.07%)		
Whether the mother and baby share the room	Yes	119.00(98.35%)	251.00(89.96%)	8.550	0.003
	No	2.00(1.65%)	28.00(10.04%)		
Whether to attend breastfeeding antenatal classes	Yes	89.00(73.55%)	207.00(74.19%)	0.018	0.890
	No	32.00(26.45%)	72.00(25.81%)		
Childbirth satisfaction	Extremely satisfied	58.00(47.93%)	119.00(42.65%)	–	0.860
	Satisfied	51.00(42.15%)	127.00(45.52%)		
	General satisfaction	11.00(9.09%)	29.00(10.39%)		
	Dissatisfied	1.00(0.83%)	2.00(0.72%)		
	Extremely dissatisfied	0.00(0.00%)	2.00(0.72%)		
Whether it has been promoted by milk powder	Yes	47.00(38.84%)	106.00(37.99%)	0.026	0.870
	No	74.00(61.16%)	173.00(62.01%)		
Whether there are breast-feeding related diseases	Yes	8.00(6.61%)	11.00(3.94%)	1.330	0.250
	No	113.00(93.39%)	268.00(96.06%)		

### 3.2 Current status and correlation between breastfeeding knowledge, breastfeeding family support, and breastfeeding self-efficacy

The mean scores of the breastfeeding knowledge questionnaire and its subscales—breastfeeding skills and breastfeeding benefits—were 11.34 (SD = 3.87), 4.20 (SD = 1.34), and 7.14 (SD = 2.99), respectively. The mean scores of breastfeeding family support and its subscales—psychological support and behavioral support—were 3.01 (SD = 0.46), 2.97 (SD = 0.49), and 3.18 (SD = 0.71), respectively. Mothers reported an intermediate level of family support. The mean scores of breastfeeding self-efficacy and its subscales—technical dimension and intrapersonal thoughts dimension—were 45.84 (SD = 15.94), 29.29 (SD = 10.11), and 16.54 (SD = 6.04), respectively. Mothers reported a low to intermediate level of self-efficacy (Table 2).

**Table 2:** Current status and correlation between breastfeeding knowledge, breastfeeding family support, and breastfeeding self-efficacy.

Variables	Mean ± SD	Breastfeeding benefits	Breastfeeding skills	Behavioral support	Psychological support	Intrapersonal thoughts	Technique
Breastfeeding benefits	7.14 ± 2.99	1					
Breastfeeding skills	4.20 ± 1.34	0.533**	1				
BKQ	11.34 ± 3.87						
Behavioral support	3.18 ± 0.71	0.188**	0.197**	1			
Psychological support	2.97 ± 0.49	0.345**	0.369**	0.327**	1		
BFSQ	3.01 ± 0.46						
Intrapersonal thoughts	16.54 ± 6.04	0.310**	0.316**	0.285**	0.579**	1	
Technique	29.29 ± 10.11	0.188**	0.282**	0.107*	0.526**	0.582**	1
BSEF-SF	45.84 ± 15.94						

(1) BKQ, Breastfeeding knowledge questionnaire; (2) BFSQ, Breastfeeding Family Support Questionnaire; (3) BSES, Breastfeeding Self-Efficacy Scale-Short Form; (4) *correlation is significant at the 0.05 level; (5) **correlation is significant at the 0.01 level.

This correlation matrix includes exclusively the core constructs of the COM-B model and the primary outcome variable. Demographic characteristics, which are detailed in Table 1, were excluded from this matrix to maintain the theoretical focus but were adjusted for in the subsequent multivariable analyses.

The normality test results indicated that the scores for Breastfeeding Knowledge, Breastfeeding Family Support, and Breastfeeding Self-Efficacy were all normally distributed. A Pearson correlation analysis was conducted to examine the relationships among these three variables. As presented in Table 2, the findings demonstrate significant positive correlations among all three factors.

### 3.3 Factors influencing exclusive breastfeeding behavior at 6 months postpartum

Candidate variables for the multivariable logistic regression were selected based on their established clinical relevance and previous literature, rather than relying solely on preliminary univariate significance. This comprehensive approach ensures rigorous adjustment for potential confounders and prevents omitted variable bias. Prior to running the model, multicollinearity was assessed. All Variance Inflation Factor (VIF) values were strictly below 3 (Supplementary Tables S2), indicating no severe multicollinearity issues. Subsequently, all selected variables were incorporated into the binary logistic regression model. After adjusting for potential confounders, the analysis identified two significant, positive predictors of exclusive breastfeeding behaviors: breastfeeding family support (*B* = 1.646, *p* < 0.001) and breastfeeding self-efficacy (*B* = 0.099, *p* < 0.001). These results are detailed in Table 3.

**Table 3:** Binary logistic regression analysis of factors associated with feeding pattern.

Variables	*B*	SE	Wald χ^2^	*P*	OR	95%CI
Constant	−11.859	1.620	53.620	0.000	<0.001	–
Breastfeeding family support questionnaire	1.646	0.425	15.009	0.000	5.186	2.255 ~ 11.924
Breastfeeding self-efficacy Scale-short form	0.099	0.014	49.814	0.000	1.104	1.074 ~ 1.135

B, regression coefficient; SE, standard error; OR, odds ratio; CI, confidence interval.

### 3.4 Structural model of maternal capability, opportunity, and motivation factors influencing exclusive breastfeeding behavior at 6 months postpartum

Several socio-demographic variables were significant in preliminary analyses. However, they were excluded from the final PLS-SEM model. The primary aim of the SEM was to test the theoretical behavioral pathways of the COM-B framework. Including numerous demographic covariates would overly complicate the structural model and reduce statistical power.

**Figure 2:**
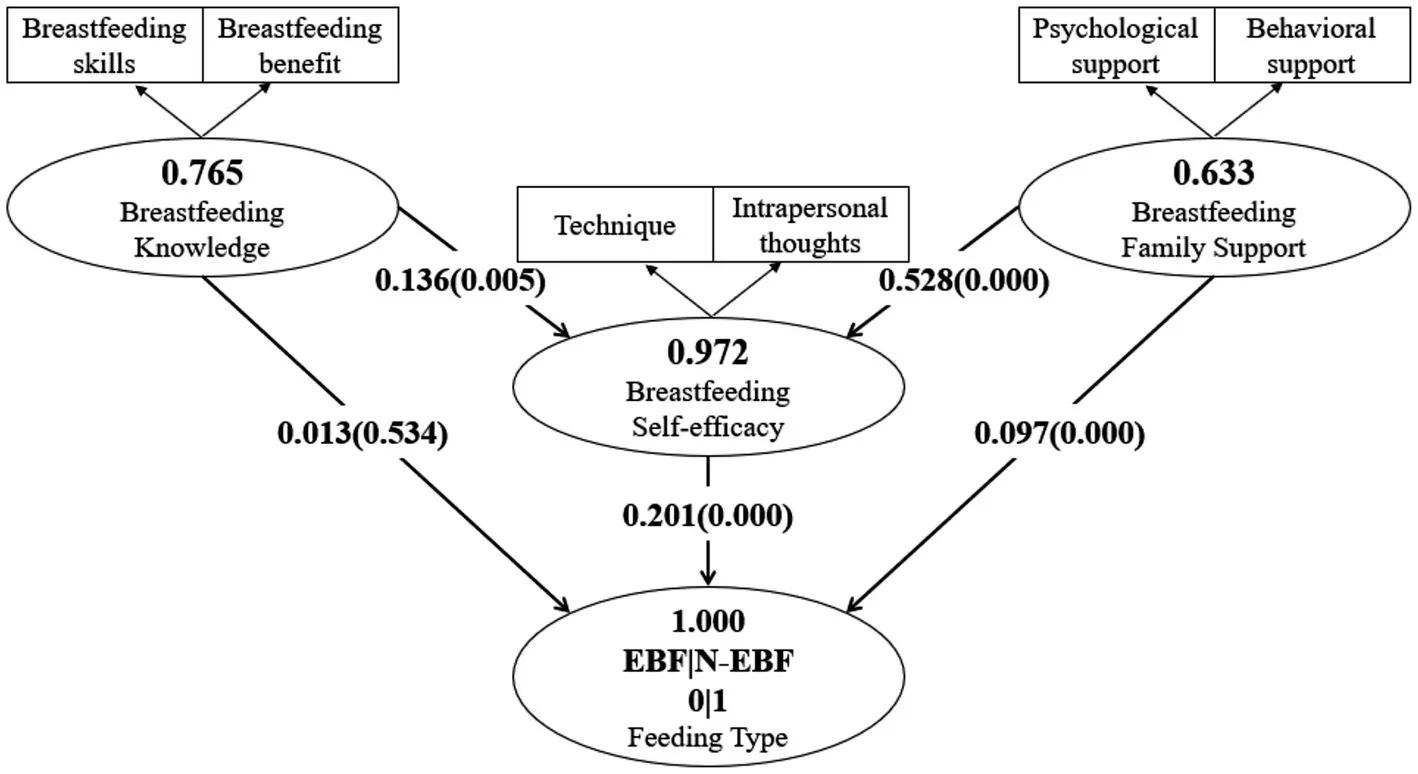
The influence pathway of exclusive breastfeeding behavior in the postpartum mothers at 6 months.

The research methodology incorporated difference analysis for categorical variables and employed Partial Least Squares Structural Equation Modeling (PLS-SEM) using SmartPLS software to examine relationships among latent variables. Path coefficient analysis revealed significant associations (*p* < 0.05) across all hypothesized relationships, with the exception of the non-significant link between breastfeeding knowledge and infant feeding patterns (*p* = 0.534), as detailed in Table 4. The structural model demonstrated that both breastfeeding family support (*B* = 0.097) and breastfeeding self-efficacy (*B* = 0.201) exerted significant positive effects on exclusive breastfeeding behavior at 6 months postpartum. Furthermore, breastfeeding knowledge (*B* = 0.136) and breastfeeding family support (*B* = 0.528) were identified as significant positive predictors of breastfeeding self-efficacy. The mediating effect of breastfeeding self-efficacy was examined using the product coefficient method with Bootstrap sampling (Table 4). The analysis revealed that breastfeeding self-efficacy significantly mediated the relationship between both breastfeeding knowledge and family support with exclusive breastfeeding behavior at 6 months postpartum (*p* < 0.05). When considering the combined direct and indirect effects, breastfeeding knowledge (*B* = 0.136, *p* < 0.05) and breastfeeding family support (*B* = 0.528, *p* < 0.05) demonstrated significant positive effects on breastfeeding self-efficacy. Furthermore, both breastfeeding family support (*B* = 0.204, *p* < 0.05) and maternal self-efficacy (*B* = 0.201, *p* < 0.05) were identified as significant predictors of exclusive breastfeeding behavior, as illustrated in Figure 2.

**Table 4:** The total effects, direct effects, and indirect effects of every path in this model.

Estimate	Point estimate	Boot SE	Boot 95%CI		*T*	*p-*value
			LLCI	ULCI		
Total effects						
BFSQ - > BSES	0.528	0.530	0.451	0.599	14.016	<0.001
BFSQ - > EBF	0.204	0.204	0.160	0.244	9.541	<0.001
BKQ - > BSES	0.136	0.136	0.039	0.228	2.811	0.005
BKQ - > EBF	0.040	0.041	−0.001	0.083	1.858	0.063
BSES - > EBF	0.201	0.201	0.157	0.245	8.926	<0.001
Direct effects						
BFSQ - > BSES	0.528	0.530	0.454	0.601	14.016	<0.001
BFSQ - > EBF	0.097	0.097	0.051	0.144	4.017	<0.001
BKQ - > BSES	0.136	0.136	0.041	0.229	2.811	0.005
BKQ - > EBF	0.013	0.013	−0.029	0.054	0.622	0.534
BSES - > EBF	0.201	0.201	0.157	0.245	8.926	<0.001
Indirect effects						
BFSQ - > BSES - > EBF	0.106	0.107	0.081	0.137	7.451	<0.001
BKQ - > BSES - > EBF	0.027	0.027	0.008	0.049	2.628	0.009

(1) BKQ, Breastfeeding knowledge questionnaire; (2) BFSQ, Breastfeeding Family Support Questionnaire; (3) BSES, Breastfeeding Self-Efficacy Scale-Short Form; (4) EBF, Exclusive Breastfeeding.

In Partial Least Squares Structural Equation Modeling (PLS-SEM), the coefficient of determination (R^2^) serves as a key indicator of predictive power, representing the proportion of variance explained in each endogenous latent variable. Generally, higher R^2^ values indicate greater predictive accuracy of the model. The current PLS-SEM model demonstrated an R^2^ value of 0.357 and an adjusted R^2^ of 0.355 for exclusive breastfeeding behavior (Table 5). These results suggest that while breastfeeding knowledge, family support, and self-efficacy collectively explain approximately 35.7% of the variance in exclusive breastfeeding behavior, additional unmeasured factors may contribute to the remaining variance in this outcome (Table 6).

**Table 5:** Self-efficacy and exclusive breastfeeding behavior of *R*^2^ in the postpartum mothers at 6 months.

Variables	O	STDEV	T (|O/STDEV|)	*P*
*R* ^2^				
BSES	0.357	0.037	9.589	<0.001
EBF/N-EBF	0.360	0.035	10.314	<0.001
*R*^2^-adj				
BSES	0.354	0.037	9.455	<0.001
EBF/N-EBF	0.355	0.035	10.099	<0.001

(1) *R*^2^, the coefficient of determination; (2) BSES, Breastfeeding Self-Efficacy Scale-Short Form; (3) O, Coefficient; (4) STDEV, Standard Deviation; (5) T, t-statistic; (6) P, *P*-value; (7) EBF/N-EBF, Exclusive breastfeeding/No Exclusive breastfeeding.

**Table 6:** Goodness-of-fit assessment of the PLS-SEM model for exclusive breastfeeding behavior in postpartum mothers at 6 months.

Fit indices	Saturated model	Estimation model
SRMR	0.031	0.108
d_ULS	0.028	0.329
d_G	0.014	0.062
Chi square value	29.559	109.975
NFI	0.981	0.929

(1) SRMR, Standardized Root Mean Square Residual; (2) d_ULS, the squared Euclidean distance; (3) d_G, the geodesic distance; (4) NFI, Normed Fit Index.

### 3.5 Model fit indices of PLS-SEM for exclusive breastfeeding behavior at 6 months postpartum

Before testing the structural relationships, we examined the collinearity among the predictor constructs; as shown in Supplementary Table S2, all inner Variance Inflation Factor (VIF) values ranged from 1.000 to 1.680, which are well below the conservative threshold of 3, indicating that collinearity is not a critical issue in our structural model. To evaluate the measurement model, we assessed internal consistency reliability, convergent validity, and discriminant validity (Supplementary Table S1). The BSES construct demonstrated robust reliability and validity (CR = 0.969; AVE = 0.695). For the BKQ and BFSQ constructs, although the AVE values (0.302 and 0.285, respectively) were below the traditional 0.50 threshold, their Composite Reliability (CR) values were 0.873 and 0.747, both exceeding the acceptable 0.60 threshold. According to (56), convergent validity is still considered adequate in this scenario, so we retained all items to preserve content validity, while excluding metrics for single-indicator constructs as they are mathematically fixed at 1.000. Additionally, we evaluated discriminant validity using the Heterotrait-Monotrait (HTMT) ratio (Supplementary Table S3). All HTMT values between the core constructs were below the conservative threshold of 0.85, and the 95% confidence intervals derived from 5,000 bootstrapping resamples did not include the value of 1, jointly confirming the discriminant validity of the constructs. Finally, we assessed the overall model fit using multiple indices (Table 5). The model yielded a Standardized Root Mean Square Residual (SRMR) value of 0.108, which, despite exceeding the strict < 0.08 threshold, is considered adequate for exploratory PLS-SEM models. Coupled with satisfactory values for other fit indices such as the squared Euclidean distance (d-ULS), geodesic distance (d-G), and Normed Fit Index (NFI), these results provide an acceptable baseline for our hypothesized structural model, especially given that PLS-SEM is primarily a prediction-oriented approach rather than a strict model-fitting technique.

## 4 Discussion

While the American Academy of Pediatrics emphasizes the unique benefits of human milk (40), maintaining exclusive breastfeeding (EBF) remains a significant public health challenge. In this multicenter study, the EBF rate at 6 months postpartum was 30.25%. This is considerably lower than the 50% target set by the “Healthy China 2030” initiative. Although mothers exhibited moderate levels of breastfeeding knowledge, family support, and self-efficacy, this relatively low EBF rate highlights a clear gap between feeding intention and actual behavior.

Using the COM-B model, our structural equation analysis explored the mechanisms behind this gap. The core finding is the key role of Motivation (breastfeeding self-efficacy). It is not only the strongest direct predictor of EBF but also a crucial mediator. Notably, Capability (breastfeeding knowledge) did not directly influence exclusive breastfeeding behavior. This suggests that theoretical knowledge alone is insufficient to drive behavior change unless it is supported by internal confidence (41). Conversely, Opportunity (family support) showed a strong two-fold effect: it directly promoted EBF by reducing practical barriers, and indirectly encouraged the behavior by enhancing maternal self-efficacy.

In this study, the exclusive breastfeeding (EBF) rate was 30.25%. This rate is lower than the 2022 Chinese national average of 36.2% (34), the global average of 48.0% (42) and the WHO target of 70%. These findings highlight the need for targeted interventions based on the COM-B model. Furthermore, this low EBF rate likely results from multiple interconnected barriers to breastfeeding, rather than a lack of maternal willingness.

Clinically, many mothers experience perceived insufficient milk (PIM) and lactation complications (43, 44), which often led to early formula supplementation. Within the COM-B framework, these clinical challenges can reduce both maternal physical Capability and Motivation (such as self-efficacy). Furthermore, working mothers in Southeast China often face a lack of physical Opportunity upon returning to work. Inadequate workplace support and limited lactation breaks can negatively affect EBF continuation (45). Given these physical and environmental barriers, our findings indicate that family support (social Opportunity) and self-efficacy (Motivation) are significant positive factors. They may help mothers maintain EBF when facing these practical challenges.

Regarding the Opportunity dimension of the COM-B model, our findings align with previous studies (46, 47) indicating that family members’ attitudes influence maternal breastfeeding choices. Specifically, family encouragement is positively associated with the adoption and continuation of EBF. This suggests that family support (Social Opportunity) is a key factor in shaping maternal feeding decisions. Therefore, clinical interventions may benefit from a family-centered approach. Providing appropriate breastfeeding education to key family members could help create a supportive home environment for EBF.

Additionally, early physical Opportunity in the hospital is important for EBF initiation and continuation. Consistent with previous studies (48), our results indicate that rooming-in is positively associated with EBF outcomes. Rooming-in minimizes mother-infant separation and facilitates early skin-to-skin contact. This practice may promote early milk production (49) (Capability) and improve maternal confidence (Motivation). These findings highlight the value of minimizing unnecessary neonatal unit admissions. When mother-infant separation is unavoidable, providing practical support—such as breast pumps and lactation counseling—may help mothers maintain milk supply and prepare for future direct breastfeeding.

The structural equation model (SEM) results indicate that breastfeeding self-efficacy (Motivation, H3 supported) mediates the relationship between capability, opportunity, and exclusive breastfeeding (EBF) behavior.

Regarding maternal Capability, theoretical breastfeeding knowledge was not directly associated with EBF behavior (H1 rejected). While previous studies have emphasized the direct role of knowledge (23, 50), this finding may reflect the “cognition-behavior gap” (51). Knowledge may serve primarily as a cognitive foundation rather than an independent driver of behavior. However, consistent with our mediation hypothesis (H4 supported), knowledge was positively associated with self-efficacy. This suggests that acquiring breastfeeding knowledge may improve maternal confidence in managing challenges, thereby indirectly relating to EBF behavior through enhanced self-efficacy (52, 53).

Furthermore, family support (Opportunity) was positively associated with EBF behavior. Supporting H2 and H5, family support demonstrated both a direct association with EBF and an indirect association through maternal self-efficacy. This finding is particularly relevant in the Chinese cultural context, where multigenerational living and shared childcare are common (54). Family members often provide practical and emotional assistance (55), which may help alleviate postpartum stress and support maternal self-efficacy.

Our findings highlight the role of family support in the Chinese context. However, alternative perspectives exist. Rollins et al. (18) found that structural factors, such as maternity leave and workplace policies, primarily determine breastfeeding duration. These factors can outweigh informal family support. In societies with different welfare systems, professional support may be more influential than extended family involvement. Therefore, the effect of family support observed in this study is likely context-dependent. This suggests that the relative weights of COM-B components can vary across different socio-cultural environments.

In conclusion, health education focusing primarily on knowledge transfer may not be sufficient to promote exclusive breastfeeding. Clinical practices may benefit from a family-centered approach. Future interventions could combine practical skills training with family involvement to support maternal breastfeeding self-efficacy and improve EBF rates.

## 5 Limitation

Several limitations should be considered when interpreting these findings. First, the cross-sectional design precludes establishing causal relationships among the COM-B constructs and EBF behaviors. Second, the use of convenience sampling from a single district in Southeast China may introduce selection bias and reduce the representativeness of the study population. Participants might differ from the broader population regarding healthcare access or socio-economic status, which limits generalizability. Furthermore, while key demographics like maternal education were controlled for, certain sociodemographic and cultural factors were not assessed. Specifically, data regarding marital status, religious beliefs, and complex family structures—such as single-parent households or conceptions via assisted reproductive technologies—were not collected. Because these factors can significantly influence breastfeeding support and practices, their omission limits our ability to fully contextualize the findings. Third, data were collected via self-reported online questionnaires. The measurement of EBF relied on a 24-h recall method, which involves potential recall bias. Additionally, due to societal expectations regarding breastfeeding, social desirability bias might lead mothers to unintentionally overestimate their EBF practices and self-efficacy levels. Finally, while the COM-B model provided a useful framework, the study did not capture all potentially relevant dimensions. Specifically, workplace-related environmental opportunities and formal lactation policies were unmeasured, despite their known importance for EBF continuation. The potential for residual confounding also remains, as unmeasured clinical variables—such as maternal postpartum depression and infant health conditions—could serve as alternative explanations for EBF behaviors outside the assessed COM-B constructs. Future longitudinal studies should incorporate these broader environmental and clinical factors to further validate our findings.

## 6 Conclusion

This study highlights that exclusive breastfeeding (EBF) rates at 6 months postpartum remain suboptimal. The findings indicate that family support and breastfeeding knowledge positively influence EBF behavior. Importantly, maternal self-efficacy serves as a central mediator connecting these factors to actual feeding practices. Family support also directly promotes EBF. Consequently, future EBF interventions should move beyond standard maternal education. Intervention development should adopt a family-centered approach to enhance collective support. Furthermore, clinical programs should prioritize building maternal self-efficacy through dynamic assessments, practical training, and peer support networks.

## Data Availability

The datasets presented in this article are not readily available because the datasets used and/or analyzed during the current study available from the corresponding author on reasonable request. Requests to access the datasets should be directed to Shengbin Guo, Gsb13774518537@163.com.
