# Supplementary material for: Decision-making process of exclusive breastfeeding at 6 months postpartum based on the COM-B model: a multicenter, cross-sectional study in Southeast China
Source: Front Public Health. 2026 Sep 4;14:1859936. doi: 10.3389/fpubh.2026.1859936 (PMC13585719; doi:10.3389/fpubh.2026.1859936)
Supplement: Supplementary file 1 [file Supplementary_file_1.docx]

**Supplementary Tables S1: Construct Reliability and Convergent Validity**

| **Construct** | **Cronbach's Alpha** | **rho_A** | **Composite Reliability (CR)** | **Average Variance Extracted (AVE)** |
| --- | --- | --- | --- | --- |
| BFSQ | 0.614 | 0.685 | 0.747 | 0.285 |
| BKQ | 0.845 | 0.859 | 0.873 | 0.302 |
| BSES | 0.966 | 0.968 | 0.969 | 0.695 |

Abbreviation:(1) BKQ: Breastfeeding knowledge questionnaire;(2) BFSQ: Breastfeeding Family Support Questionnaire;(3) BSES: Breastfeeding Self-Efficacy Scale-Short Form;

**Supplementary Tables S2: Structural Model Collinearity Diagnostics (Inner VIF)**

| **Target Construct** | **Predictor Construct** | **Inner VIF** |
| --- | --- | --- |
| BSES | BFSQ | 1.216 |
|  | BKQ | 1.216 |
| EBF | BFSQ | 1.680 |
|  | BKQ | 1.234 |
|  | BSES | 1.561 |

Abbreviation:(1) BKQ: Breastfeeding knowledge questionnaire;(2) BFSQ: Breastfeeding Family Support Questionnaire;(3) BSES: Breastfeeding Self-Efficacy Scale-Short Form; (4) EBF: Exclusive Breastfeeding

### **Supplementary Tables S3: Discriminant Validity (HTMT Criterion with Bootstrapping Confidence Intervals)**

| **Construct** | **1. BFSQ** | **2. BKQ** | **3. BSES** | **4.** **EBF** |
| --- | --- | --- | --- | --- |
| 1. BFSQ | - |  |  |  |
| 2. BKQ | 0.544  [0.438, 0.616] | - |  |  |
| 3. BSES | 0.661  [0.571, 0.734] | 0.370  [0.272, 0.466] | - |  |
| 4. EBF | 0.503  [0.411, 0.583] | 0.256  [0.166, 0.347] | 0.576  [0.513, 0.636] | - |

Abbreviation:(1) BKQ: Breastfeeding knowledge questionnaire;(2) BFSQ: Breastfeeding Family Support Questionnaire;(3) BSES: Breastfeeding Self-Efficacy Scale-Short Form; (4) EBF: Exclusive Breastfeeding

Note: Values in brackets represent the 95% bias-corrected and accelerated (BCa) confidence intervals obtained through a bootstrapping procedure with 5,000 resamples. Discriminant validity is established as none of the confidence intervals include the value of 1.
